# Arrangements of Mobile Genetic Elements among Virotype E Subpopulation of *Escherichia coli* Sequence Type 131 Strains with High Antimicrobial Resistance and Virulence Gene Content

**DOI:** 10.1128/mSphere.00550-21

**Published:** 2021-08-25

**Authors:** Omid Pajand, Hamzeh Rahimi, Narges Darabi, Solaleh Roudi, Khatereh Ghassemi, Frank M. Aarestrup, Pimlapas Leekitcharoenphon

**Affiliations:** a Section for Genomic Epidemiology, National Food Institute, Technical University of Denmark, Lyngby, Denmark; b Microbiology Department, Faculty of Medicine, Semnan University of Medical Sciences, Semnan, Iran; c Department of Molecular Medicine, Biotechnology Research Center, Pasteur Institute of Iran, Tehran, Iran; d Student Research Committee, Faculty of Medicine, Semnan University of Medical Sciences, Semnan, Iran; e Imam Hossein Hospital, Semnan University of Medical Sciences, Aradan, Iran; University of California, Davis

**Keywords:** ST131, whole-genome sequencing, hemolysin, genomic island, SNP phylogeny, virulence, cgMLST, Iran, cytotoxin, virulence factors

## Abstract

Escherichia coli sequence type 131 (ST131) is known for its contribution to multidrug resistance and the worldwide spread of this clone has become a global problem. Understanding the trends among ST131 clades will help design strategies to prevent its rapid dissemination. In this study, 72 ST131 strains were subjected to comparative genomic analysis and 64 clade C strains were compared with clade C strains reported from other regions using publicly available whole-genome sequencing data. C1 (*n* = 31 [48.4%]) and C2 (*n* = 33 [%51.5]) strains had the same prevalence in our collection, and C1-M27 (*n* = 22) strains were closely related, carried a unique plasmid type (F1:A2:B20), and exhibited virotype C. Removal of 11 C2 strains with varied virotype patterns and the heterogeneous IncF type identified 22 closely related virotype E/F strains with replicon type F31/F36:A4:B1, forming what we denote as the “C2-subset.” In a global context, the C2-subset constituted a distinct cluster with international virotype E strains and harbored a genomic island, *GI-pheU*. Association of *cnf1/hlyCABD* genes with 1 to 7 mobile genetic elements, mostly IS*682*/IS*Kpn37* combination within *GI-pheU* was identified. The C2-subset accounted for excess resistance/virulence of subclade C2 relative to C1 strains. In addition, a conserved chromosomal IS*26*-mediated composite transposon (IS*15DIV*-IS*Ecp1*-*bla*_CTX-M-15_-*WbuC* cupin fold metalloprotein-Tn*2-*IS*15DIV*) was observed in the C2-subset. The local spread of the C2-subset in the hospital studied, with the carriage of higher virulence/resistance markers and a peculiar F-type plasmid, demonstrates the potential for diversification of the ST131 lineage and the emergence of subpopulations with higher survival potential to cause health care-associated outbreaks.

**IMPORTANCE**Escherichia coli sequence type 131 (ST131) is a globally dominant multidrug-resistant clone that is commonly associated with extraintestinal infections. Specific sublineages have been shown to have emerged and spread within ST131, highlighting the complex nature of ST131 epidemiology. This study systematically compared the Iranian ST131 population to those reported from other countries and found a subpopulation harboring virotype E, a homogeneous profile of plasmid Inc-F type F31/F36:A4:B1 harboring *cnf1*/hemolysin genes on the genomic island *GI-pheU*, and up to seven mobile genetic elements (MGEs) flanking *cnf1*/hemolysin virulence markers. The results of this study highlight the importance of MGEs for virulence gene acquisition and the formation of new subpopulations among pandemic clones such as E. coli ST131.

## INTRODUCTION

Escherichia coli sequence type 131 (ST131) is an example of successful multidrug-resistant pandemic clone among human pathogens (1, 2). ST131 is the most common clone among extraintestinal pathogenic E. coli (ExPEC) and shows the highest rates of fluoroquinolone (60 to 90%) and cephalosporin (40 to 80%) resistance among this pathotype (3, 4). Population genetics studies have delineated the ST131 phylogeny into three major clades (5). Clades A and B are minor subsets among ST131 clones, and clade C represents the largest clade and comprises two sublineages, C1 (*H30*R) and C2 (*H30*Rx). Clade C is uniformly fluoroquinolone resistant due to conserved replacement mutations in *gyrA* and *parC* and mostly shows cefotaxime/ceftazidime-resistant phenotypes due to producing of *bla*_CTX-M-15_ and *bla*_CTX-M-27_ enzymes. C1 subclade is mainly associated with carriage of *bla*_CTX-M-27_ or *bla*_CTX-M-14_. In contrast, C2 subclade which is subdivided from C1 based on specific a single nucleotide polymorphism (SNP) at *fimH30*, mostly harbor *bla*_CTX-M-15_ and represents the dominant population among clade C (6). Compared to the highly conserved core genome, there is a wide plasticity in the accessory genome of ST131 resulting in appearance of different virotypes and plasmid content (7).

Based on genome sequence analyses of ST131 isolates, the major determinants of this clone have been partially characterized (8–10). The harboring of plasmids belonging to incompatibility (Inc) groups with F replicons that acquire resistance genes and spread rapidly is one of ST131 prominence. The most common pMLST types identified among multidrug resistant C1 and C2 subclades are F1:A2:B20 and F2:A1:B-, respectively (5, 11–13). Virulence gene content plays the other preponderant role in the evolution of this clone. Several studies have shown that few virulence genes are consistent among ST131 strains and there is variation in the repertoire of virulence genes (14). In this regard, the emergence of new virotypes within the C2-subclade demonstrates the evolving nature of this subpopulation, particularly with a recent report of C2/*H*30Rx strains harboring *cnf1*, *tia/hek*, and *hlyCABD* genes from Singapore (15). The importance of virulence gene acquisition is underscored by the “perfect storm” theory first proposed by Ben Zakour et al. (14), which states that this acquisition is followed by the development of antibiotic resistance in a pandemic clone such as E. coli ST131. The acquisition of some virulence genes such as autotransporter genes *agn-43* and *sat*, the gene cluster encoding aerobactin biosynthesis (*iucABCD*) and the gene encoding the siderophore receptor *iutA* through the acquisition of the genomic island *GI-pheV* has been shown in clade C strains (14). In our previous study of the ST131 collection, we found the occurrence of a subset under subclade C2 harboring *hlyA/cnf1* virulence genes and their association with the expression of the aminoglycoside resistance phenotype and a higher level of virulence genes (16). This motivated us to conduct a whole-genome analysis of population structure, focusing on the *cnf1/hlyCABD*-bearing subset in the context of a local hospital and global collections to further our understanding of the evolving nature of the pandemic ST131 clone. Analysis of the *cnf1/hlyCABD* carrying strains reveals that they are carried by acquisition of a horizontally transmitted genomic island, *GI-pheU*. We also describe the association of these virulence genes with up to seven MGEs on positive contigs.

## RESULTS

### Highest resistance rate was observed against cefotaxime.

In this study, we focused on the genetic profiles of 72 ST131 E. coli strains isolated during March 2015 to September 2016 (19 months). Of the 72 patients with an age range of 13 to 97 years, 31 (43.1%) were male. Data regarding the sample source, MDR phenotype, and patient’s gender in terms of clade classification are presented in Table 1. Antibiotic susceptibility testing revealed the highest susceptibility rates against imipenem (97.2%), meropenem (98.6%), amikacin (88.9%), and ertapenem (87.5%). The highest resistance rates were observed against cefotaxime (*n* = 67, 93.1%). All eight clade A strains were susceptible to fluoroquinolone.

**TABLE 1:** Demographic characteristics of studied ST131 isolates

Clade or subclade (total no. of isolates)	No. (%) of isolates from females	Source of isolation (*n*)^*a*^	No. (%) of isolates^*b*^	
			MDR	ExPEC
Clade A (8)	6 (75)	Respiratory (1), UC (7)	6 (75)	4 (50)
Subclade C1 (31)	17 (54.8)	Respiratory (5), UC (24), wound (2)	28 (90.3)	29 (93.5)
Subclade C2 (33)	18 (54.5)	UC (31), wound (2)	32 (97)	33 (100)

a UC, urine culture; *n*, number of isolates.

b MDR, multidrug resistant; ExPEC, extraintestinal pathogenic E. coli.

### Equal distribution of subclade C1 and C2 among collected strains.

**FIG 1:**
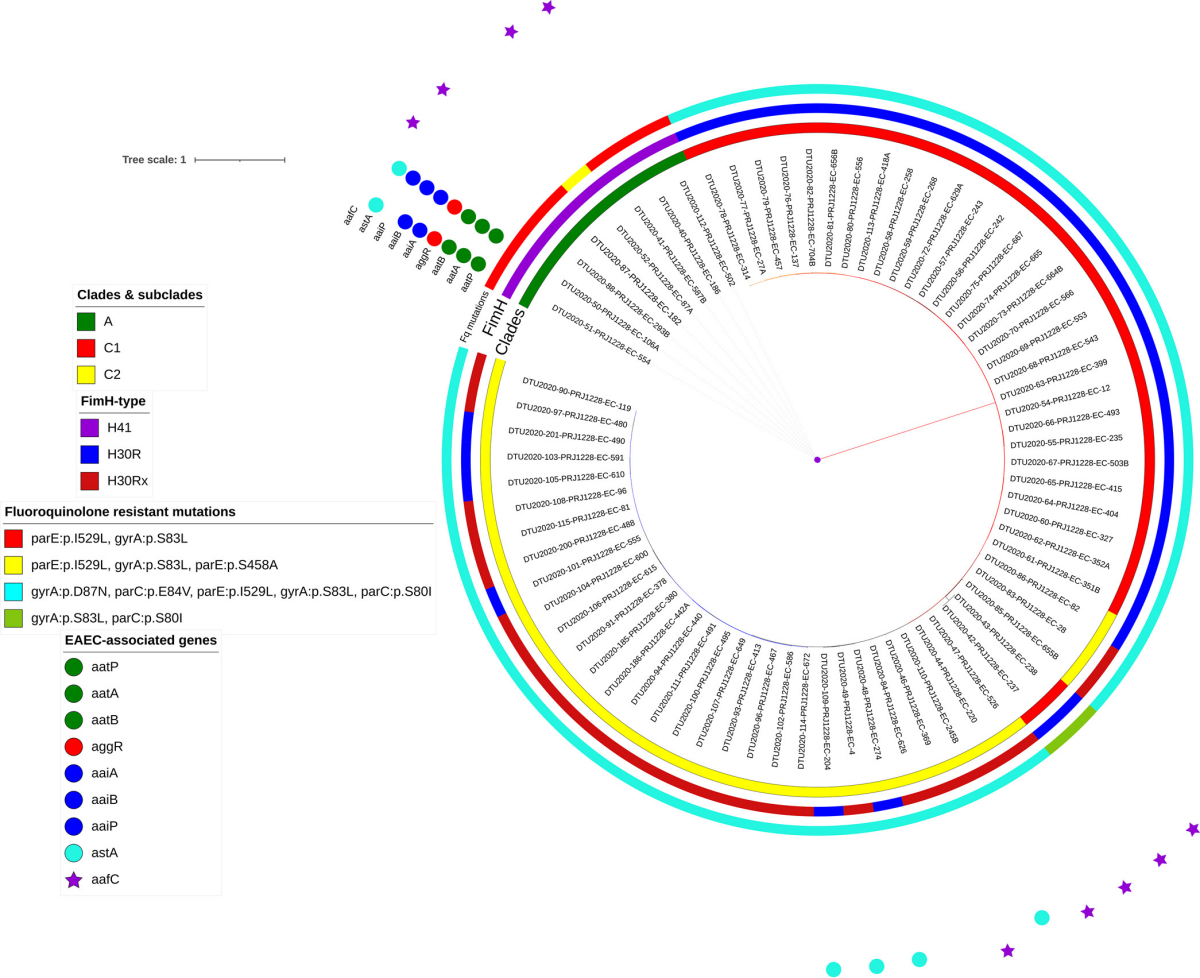
Phylogenetic reconstruction of *n* = 72 collected ST131 strains. Maximum-likelihood phylogeny of *n* = 72 ST131 showed two main clades and C (*n* = 64), with two common subclades in C: C1 (*n* = 31) and C2 (*n* = 33). The midpoint-rooted phylogram was constructed with PhyML from the chromosome-wide SNPs arising by mutation and visualized with iTol. Allelic profiling of *fimH*, *gyrA-parC*, clade classification, and EAEC-associated genes is represented as colored strips around the phylogenetic tree.

Two clade A strains were characterized as EAEC. The other EAEC-associated genes, such as *aafC* and *astA*, were identified among nine and four strains, respectively. The resistance genes detected among the eight clade A strains were *bla*_TEM-1B_ (100%), *bla*_CTX-M-15_ (62.5%), *aadA5*, *aph6Id*, *aph3Ib*, *tet*(A) (62.5%), *aac3IId*, *tet*(B) (25%), and *bla*_CMY-42_ (12.5%). Mutations detected in two subunits of DNA-topoisomerase IV and DNA-gyrase genes in clade A strains were *parE*(I529L/S458A) and *gyrA*(S83L), respectively. The phylogeny of the 72 strains based on genomic alignment contained 7,074 SNPs totally. This recapitulated the clades A and C and showed that most strains were from clade C (*n* = 64, all fluoroquinolone resistant), specifically subclade C2 (*n* = 33), followed by subclade C1 (*n* = 31) and clade A. All clade A and C strains were *fimH*41 and *fimH*30, respectively (Fig. 1).

We focused our subsequent analyses on clade C strains since they represented the most common clade.

### Appearance of a subset within the C2-sublineage.

**FIG 2:**
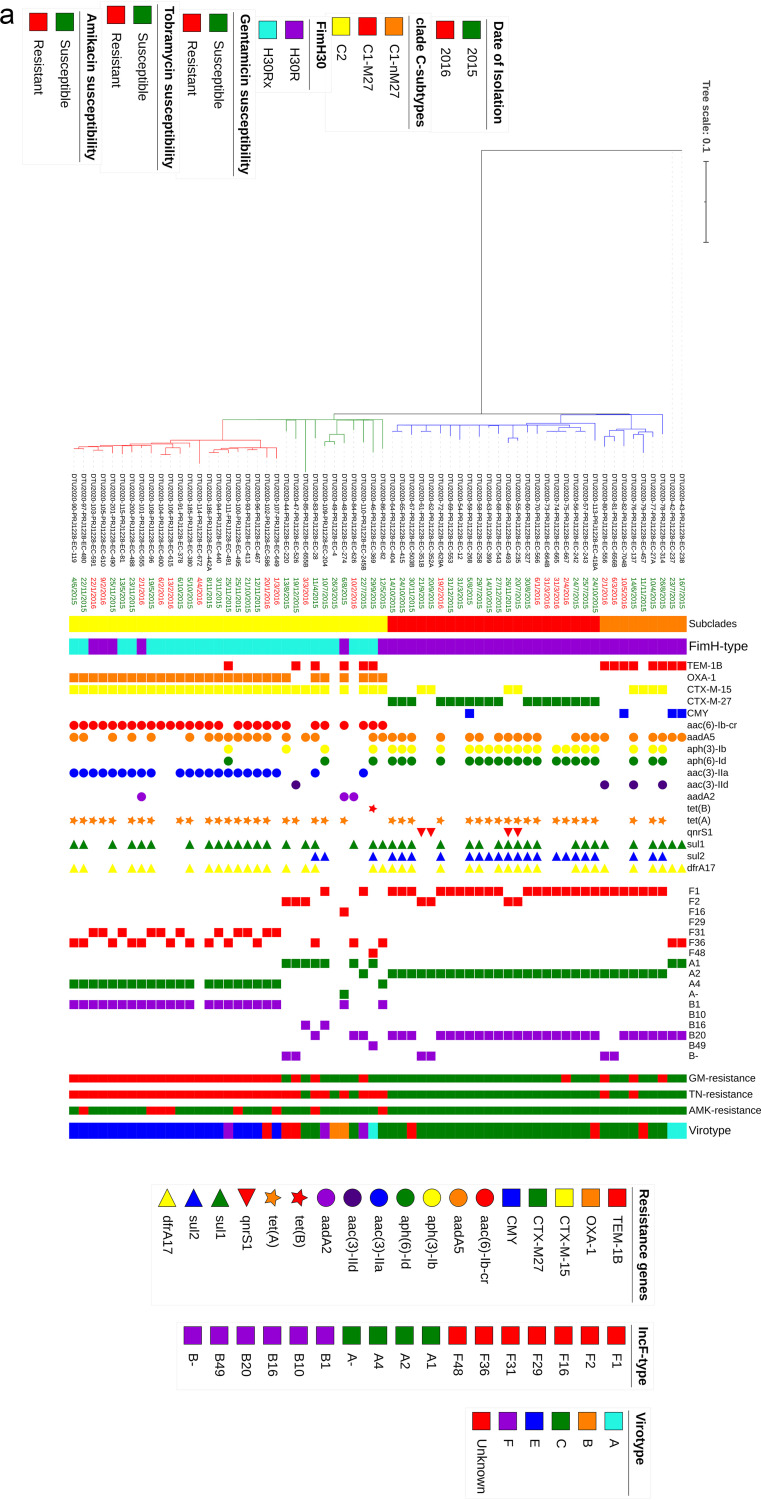

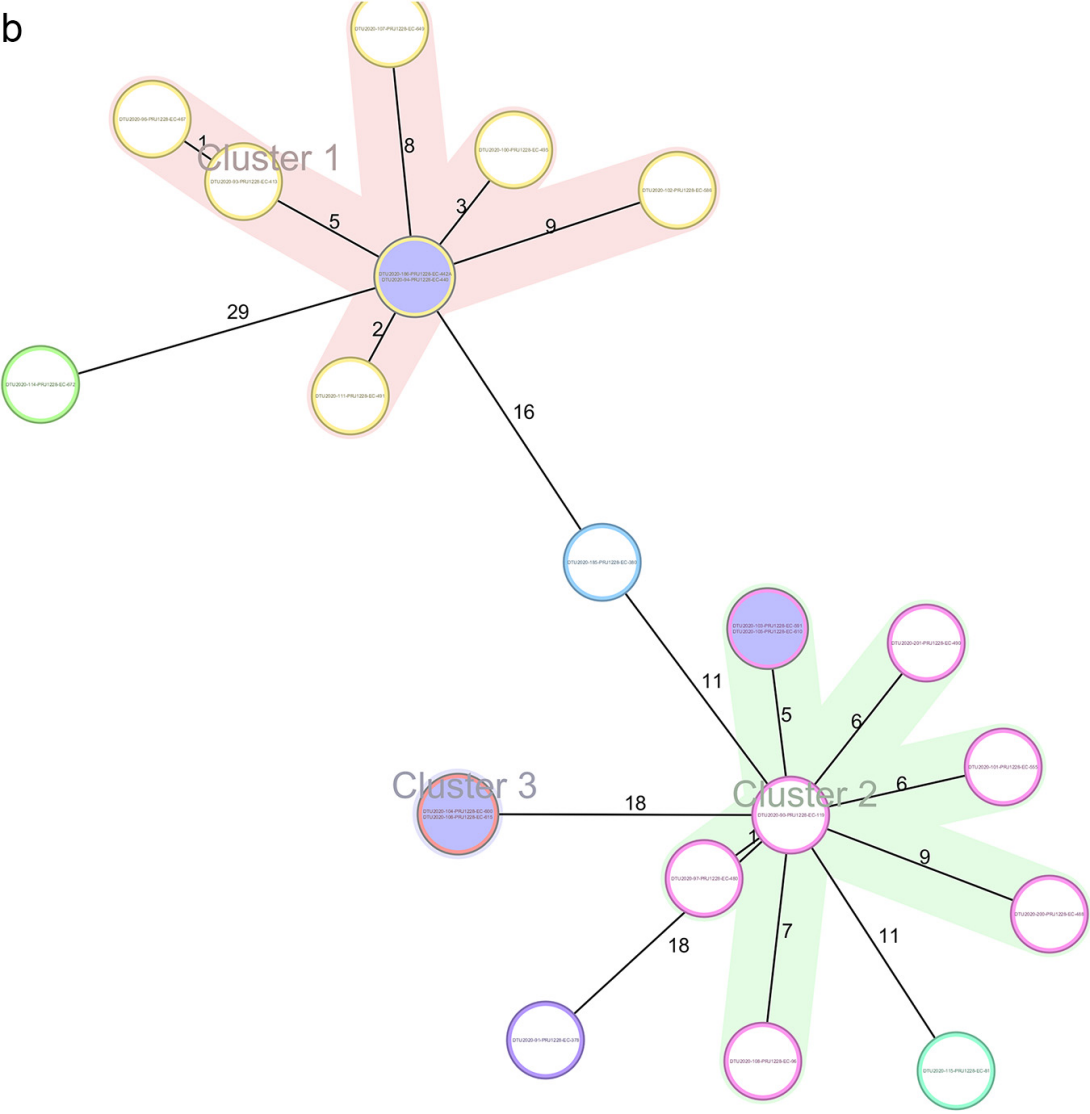
(a) Maximum-likelihood phylogeny of clade C strains. A phylogenetic reconstruction of 64 strains from clade C is shown. This image shows three common subclades in C, C1-nM27 (*n* = 9), C1-M27 (*n* = 22), and C2 (*n* = 33), where the last is divided into two groups, including C2 (*n* = 11) and C2-subset (*n* = 22). The data presented in the phylogram represent the date of isolation, C-subclade classification, the *fimH* allele type, resistance genes, the plasmid Inc-F types, aminoglycoside susceptibility patterns, and virotypes. The red branches in the phylogram represents the C2-subset strains. (b) Minimum spanning tree of 22 C2-subset isolates. The genomes were analyzed using an *ad hoc* E. coli scheme based on 2,764 targets. A maximum allelic distance of ≤10 alleles was used to define a cluster. The isolate ID numbers are shown in the bubbles, and the allelic distances between isolates are expressed on the lines connecting them. Clusters are labeled and indicated by gray zones around the included isolates.

Clade C strains were divided equally into the C1 (*n* = 31) and C2 (*n* = 33) subclades. C1 strains mainly belonged to C1-M27 (*n* = 22), and all except four strains harbored *bla*_CTX-M27_. Among the nine C1-nM27 strains, eight, four, and three strains were positive for *bla*_TEM-1B_, *bla*_CTX-M-15_, and *bla*_CMY-59_, respectively. As expected, there was a strong association between the carriage of *bla*_CTX-M-_ (CTX-M-15 and CTX-M-27) and resistance phenotypes against ceftazidime/cefepime (*P < *0.001). Aminoglycoside resistance genes *aph6Id* and *aph3Ib* were mostly detected among C1 (C1; *n* = 20, 64.5%, *P* < 0.001 for both genes); however, *aac6Ib-cr* and *aac3IIa* were mostly harbored by the C2-sublineage (Fig. 2a). The most common plasmid type among C1 was F1:A2: B20, while C2 strains showed varied plasmid replicon types. Genome alignment-based phylogeny showed 1,707 total SNP differences between clade C strains (Fig. 2a).

Interestingly, 22 of 33 C2 strains clustered closely in a subset. The leading branch to this subset had 100% bootstrap support, which suggest a monophyletic origin. Strains of this subset all carried *bla*_CTX-M-15_ and *bla*_OXA-1_, and most harbored *aac6-Ib-cr* (*n* = 21 [95.4%]) and *aac3IIa* (*n* = 20 [90.9%]). Here, we refer to these 22 strains as the “C2-subset.” Considering the plasmid replicon type, C2 strains were not uniform and harbored a mixed combination of plasmid types, and the C2-subset strains showed a homogeneous replicon pattern as F31 or F36:A4:B1, except for one strain, which was negative for IncF type plasmid (strain EC672).

A minimum spanning tree based on cgMLST of the C2-subset divided the 22 strains into three clusters, with allelic distances that ranged from 0 to 29 alleles. No differences were observed based on the distribution of resistance/virulence markers and pMLST replicon patterns between three identified clusters (Fig. 2b).

### The C2-subset harbors higher virulence markers.

**FIG 3:**
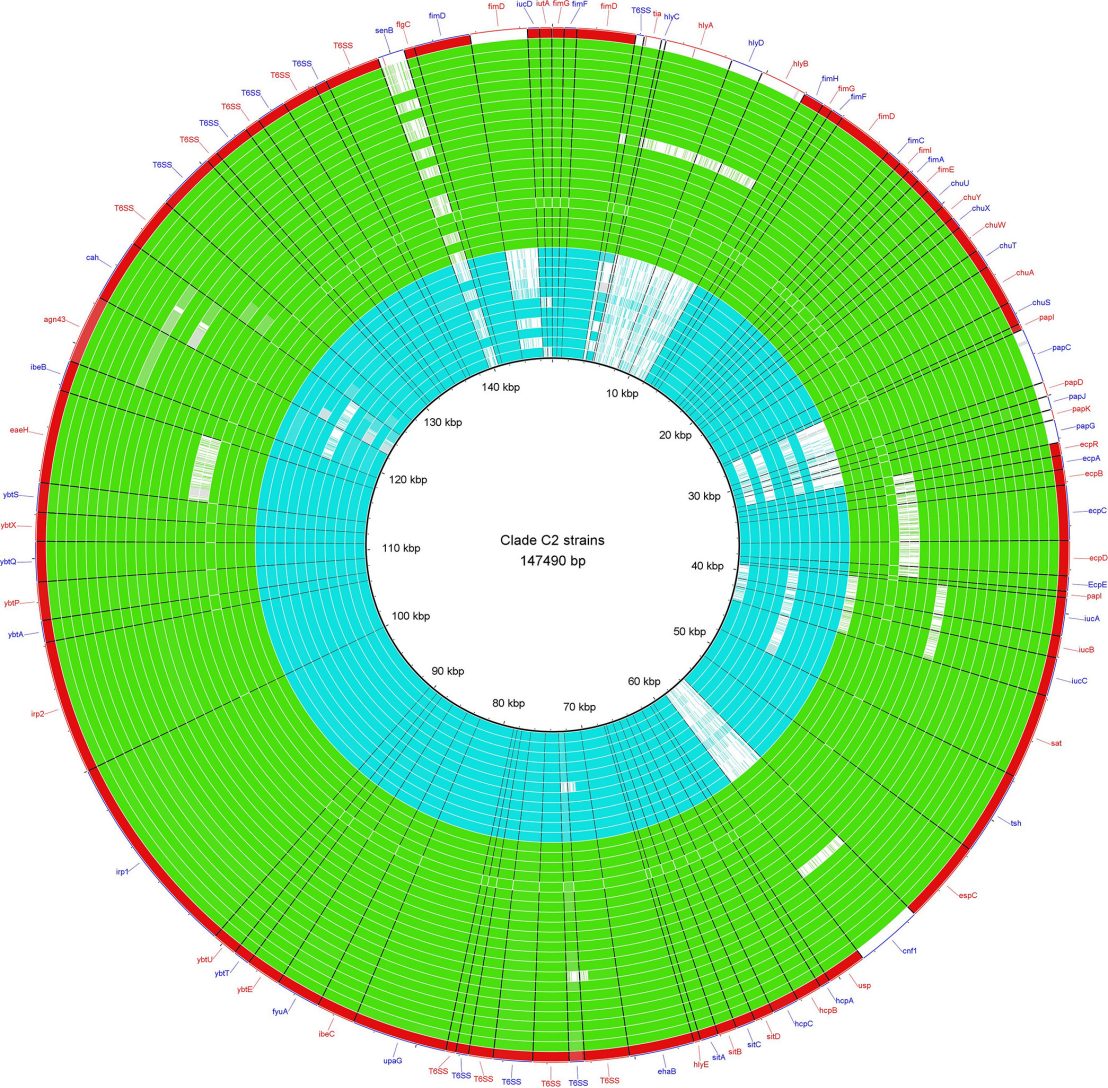
Virulence genes content of ST131 subclade C2. A BLAST Ring Image Generator (BRIG) image shows the presence of virulence factors among C2 (aqua rings) and C2-subset strains (green rings). The *FFN* format file of EC119 (one of the C2-subset) was downloaded from VFDB website, and open reading frames (ORFs) are annotated based on the VFDB results and used as reference strains to draw the image. The last ring (red) is EC958.

Toxin (*senB*, *cnf1*, and hemolysin encoding cluster [*hlyCABD*]), invasin (*tia/hek*), adhesin (*hra, papK, papD, papC,* and *papGII*), protection (*kpsmTII-k5*), and contact-dependent inhibition system (*cdiB*) (Fig. 3) genes were common among the C2 subset compared to other C2 strains. Virotyping categorized 64 strains into five virotypes, with the highest prevalence of virotype C (*n* = 30 [46.8%]) and E (*n* = 20 [31.2%]). Interestingly, virotype C was the most common pattern among subclade C1, whereas C2 strains displayed virotype heterogeneity. By focusing specifically on 22 C2-subsets, all except two strains were identified as virotype E (positive for *papC*, *papGII*, *kpsmTII-k5*, *sat*, *cnf1*, and *hlyA*) and the remaining two ones belonged to virotype F (*cnf1/hlyCABD* negative) and unknown (*kpsmTII-k5* negative). Of 64 clade C strains, 6 (9.3%) displayed an unknown virotype.

### C1-M27 and C2-subset strains differentiated into two distinct clusters in the global context.

**FIG 4:**
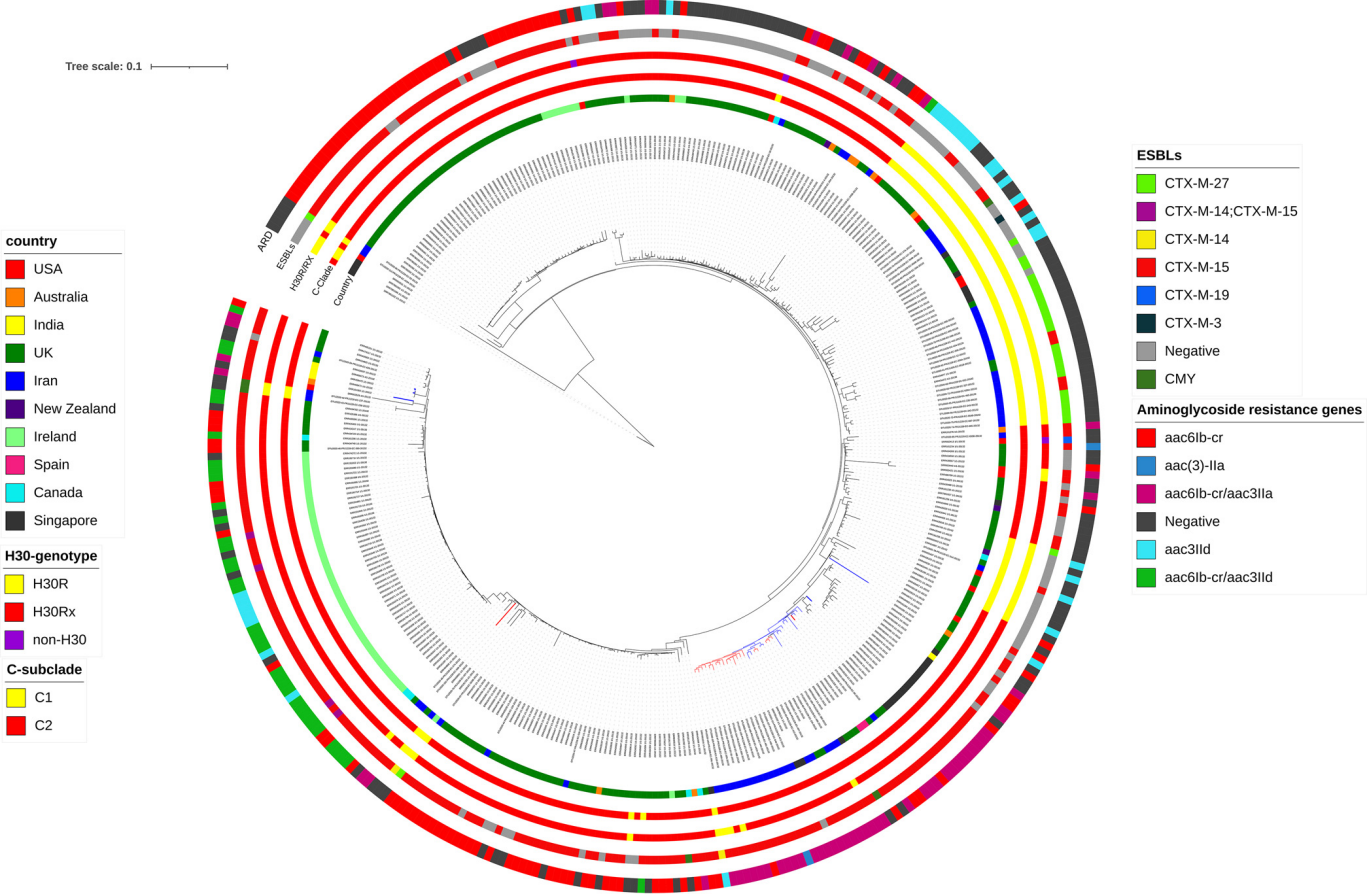
Maximum-likelihood phylogeny of clade C strains from the global ST131 collection. A phylogenetic reconstruction of 378 strains from clade C with EC958 as the reference strain is shown. This shows two common subclades in C, C1 (*n* = 76), and C2 (*n* = 302). The red branches indicate the C2-subset, and blue branches indicate the international *cnf1/hlyCABD*-positive genomes. Colored strips surrounding the phylogram represent the country of origin of each strain, clade classification, *fimH*30 allele, ESBL genes detected alone or in combination with other ESBLs, and aminoglycoside resistance determinants (ARDs) alone or in combination with other ARDs.

We analyzed the 64 clade C strains from Iran in a global context using 314 publicly available clade C whole-genome sequence data present in ENA database. From the study conducted by Ludden et al. (17), 289 genomes, including strains from C1-10, C2-7, C2-8, and C2-9, Fastbaps clusters were selected. The 25 remaining genomes were selected from a collection of recently published Singaporean clade C strains isolated from bacteremia patients. A phylogenetic tree of 378 genomes (including our 64 clade C strains) showed that Iranian isolates clustered into two groups: first, C1-M27 strains that were closely related and clustered with other international *bla*_CTX-M-27_-producing isolates, and second, a cluster including 21 of 22 C2-subset isolates that were closely grouped with 14 international genomes, all belonging to virotypes E and F. The remaining C2-subset strain that did not cluster with other *cnf1/hlyCABD*-carrying isolates was identified as unknown virotype. The 11 C2 strains were mostly diverse and distributed among different C2 clusters. We identified 20,132 SNPs by mapping of 378 genomes to EC958. The C-subclade classification was supported by *fimH*30R/Rx allelic differences, except for 11 C2 strains which were identified as *H*30R or non-*H*30. The β-lactam resistance markers, including *bla*_CTX-M-27_ and *bla*_OXA-1_, were exclusively detected among C1 and C2, respectively, while strains harboring *bla*_CTX-M-15_ (C2; *n* = 228 [75.2%], *P* < 0.001) were distributed among two C sublineages. Of the aminoglycoside resistance determinants (ARDs), *aac6Ib-cr* (C2; *n* = 211 [69.6%], *P* < 0.001) and *aac3IIa* (C2; *n* = 61 [20.1%], *P* < 0.001) were just detected among subclade C2. The other resistance markers, including *aac3IId* (C1; *n* = 19 [25.7%]), *aadA5* (C1; *n* = 26 [35.1%], *P:* 0.002), *aph3Ib* (C1; *n* = 25 [33.8%], *P* < 0.001), and *aph6Id* (C1; *n* = 25 [33.8%], *P* < 0.001), were harbored by both C subclades, with the last three were significantly detected among the C1 subclade (Fig. 4).

### The *cnf1/hlyCABD* carrying strains display similar resistance/virulence/pMLST repertoire patterns.

**FIG 5:**
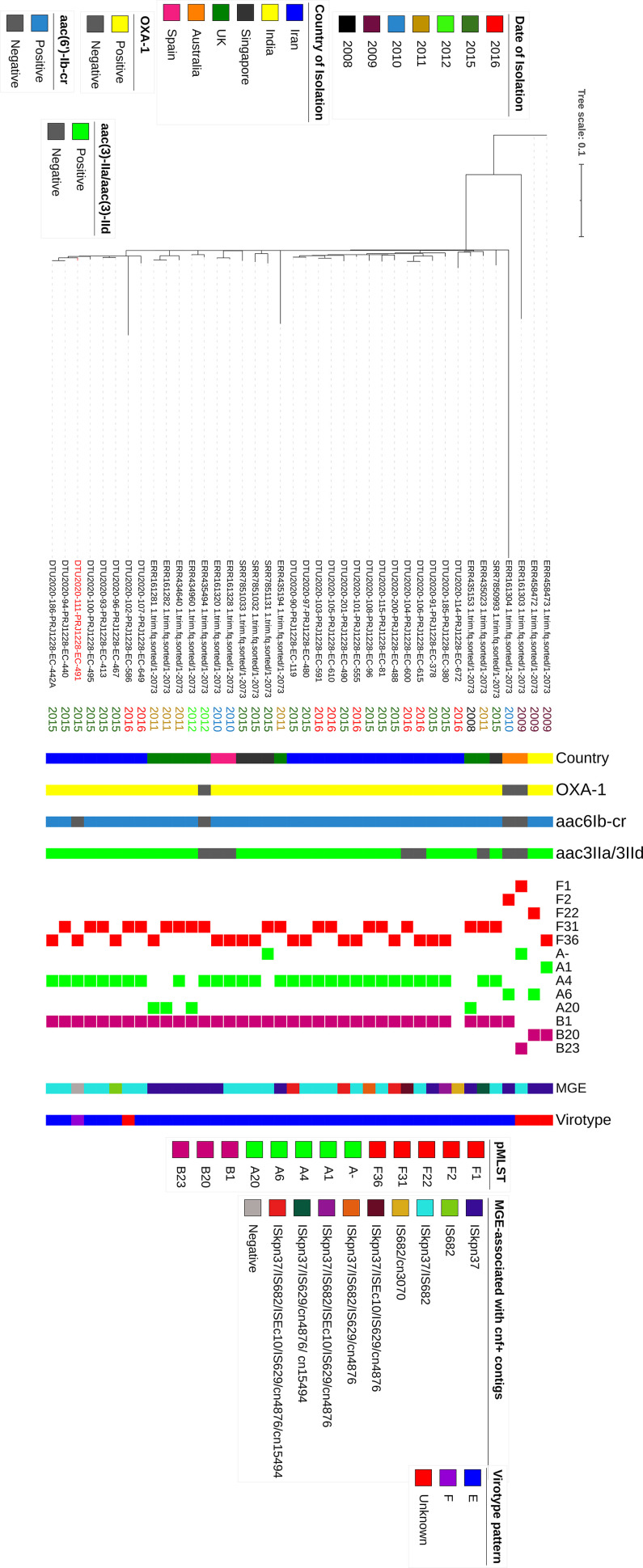
Maximum-likelihood phylogeny of the C2-subset and international *cnf1/hlyCABD*-carrying strains. A phylogenetic reconstruction of 40 strains from subclade C2 is shown. All except one strain were positive for *bla*_CTX-M-15_. C2-subsets are divided into two clusters, with international isolates distributed among them. Virotype F strain (strain 491) is indicated by a red label. The data presented in the phylogram represent the date of isolation, the country of isolation, *bla*_OXA-1_, *aac6Ib-cr*, *aac3IIa/aac3IId*, pMLST replicon types, virotypes, and MGEs associated with *cnf1/hlyCABD* genes.

The 22 C2-subset, along with 18 C2 *cnf/hlyCABD*-positive international isolates, was subjected to SNP phylogeny construction, and a total of 2,073 SNP differences were identified. The isolation dates for international isolates were from 2008 to 2015. All except one strain were *bla*_CTX-M-15_ positive and mostly harbored *aac6Ib-cr* (*n* = 36 [90%]), *bla*_OXA-1_ (*n* = 37 [92.5%]), and *aac3IIa* and *aac3IId* (*n* = 32 [80%]). The most common detected plasmid replicons among 40 strains were F31:A4:B1 and F36:A4:B1. Virotyping identified 35 strains as virotype E. Of the remaining five strains, one was identified as virotype F, and four were identified as *kpsmTII-k5* negative and categorized as unknown (Fig. 5).

### Carriage of *cnf1/hlyCABD* genes is mediated by genomic island *pheU* in association with up to seven MGEs.

**FIG 6:**
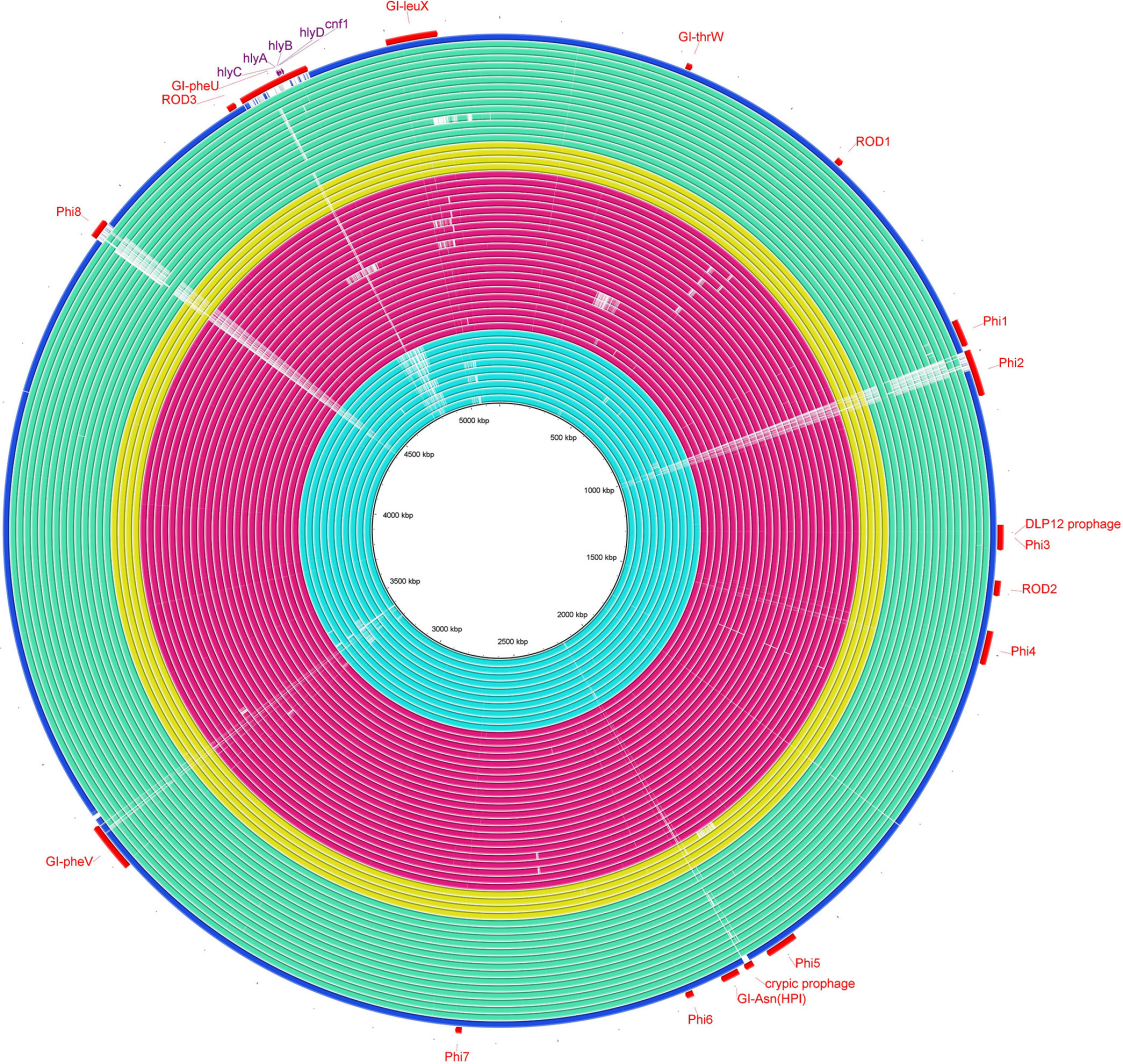
Genome similarities to the Escherichia coli ST131 S65EC genomic islands. Rings drawn by BRIG show the presence of these regions among 11 C2 (“deep sky blue” rings), 22 C2-subset (red rings), and 18 international virotype E strains (yellow and “aquamarine” rings). Colored segments indicate >70% similarity, and gray segments indicate >50% similarity by BLAST comparison between the regions of interest and each genome. The *GI-pheU* harboring *cnf1/hlyCABD* gens were detected among all except one C2-subset strain, which was negative for the mentioned virulence genes (virotype F), and also international genomes. The prophage 8 (*Phi8*) region was completely found in one C2 (EC274) and one international strain (ERR161303), and prophage 2 (*Phi2*) was partially detected among all except one international strain (ERR161303). The last ring (blue ring) indicates EC958 strain.

BRIG analysis of mobile genetic elements (MGEs) among our C2 strains (*n* = 33) and 18 *cnf/hlyCABD*-positive international isolates, with a recently sequenced strain C2/S65EC as the reference, identified a shared genomic island *GI-pheU* among *cnf1/hlyCABD*-carrying strains, which was not detected among EC958 reference strain and other C2 isolates (Fig. 6). However, conserved structure (complete identity 100%) with *GI-pheU* just was observed in four strains (all were international strains). Prophage *phi-8* was either partially or completely missing in all except for two strains (ERR161303 and EC274), and *phi-2* prophage was detected as intact in one international strain (ERR161303). Since S65EC was used as the reference strain in BRIG analysis and this strain is negative for *GI-selC* compared to EC958, we could not show *GI-selC* carriage among the studied 40 strains.

**FIG 7:**
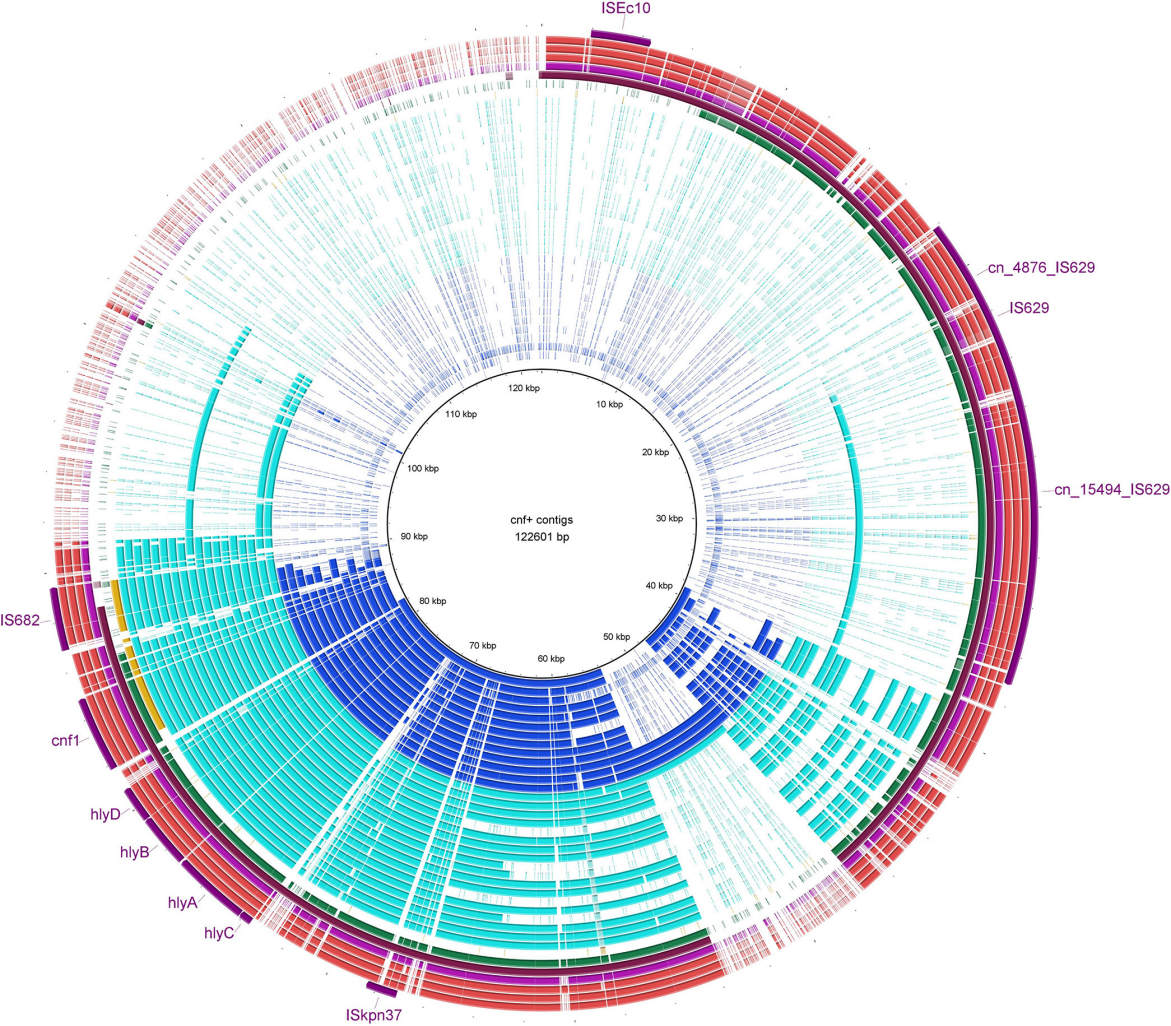
Mobile genetic elements within contigs carrying *cnf1/hlyCABD*. A BLAST Ring Image Generator (BRIG) image shows the presence of the MGEs reported in *GI-pheU* (122,601 bp) in association with *cnf1/hlyCABD* among our data set. Colored segments indicate >70% similarity, and gray segments indicate >50% similarity by BLAST comparison between the regions of interest and each contig. Blue rings indicate contigs harboring one MGE (IS*Kpn37*), aqua rings indicate contigs harboring two MGEs (IS*682* and IS*Kpn37*), yellow rings indicate strain EC672 with a truncated *cnf1*, and the last six rings are strains harboring four to six MGEs in association with *cnf1/hlyCABD*.

Focusing on contigs carrying *cnf1/hlyCABD* cluster and *GI-pheU* as the reference for BRIG analysis, we identified *cnf1/hlyCABD* carriage with the association of 1 to 7 mobile genetic elements, including IS*682*, IS*Ec16*, IS*Kpn37*, IS*629*, IS*Ec10*, and three composite transposons (cn_4876_IS*629*, cn_15494_IS*629*, and cn_3070) (Fig. 7). IS*Kpn37* was present in all except one strain which carried *IS682* in association with IS*Ec16*. The most common MGE combination patterns were IS*Kpn37* alone or along with IS*Ec16* and IS*682*, in the same direction of *cnf1/hlyCABD* genes (see Fig. S1a and b in the supplemental material). The genetic structure identified on all except two *cnf1/hlyCABD* carrying contigs was IS*Kpn37*, histidine kinase, *hlyCABD/cnf1*, “helix-turn-helix domain containing protein,” and IS*682*, of that histidine kinase and “helix-turn-helix domain containing protein” were in reversed direction. In the remaining two strains (EC467 and EC672), the truncated form of *cnf1* was identified on two contigs. The observed pattern in these two strains was in this format: ∼500 bp of *cnf1*, along with *hlyCABD* operon on one contig, and the remaining ∼2,500 bp on the other contig (see Fig. S1c and d). The carriage of MGE divided the C2-subset into two groups. Group 1 carried two MGEs, along with *cnf1/hlyCABD* genes, while group 2 strains harbored mixed combinations of MGEs (from one to seven elements) (Fig. 5). The remaining 18 public positive strains were associated with up to four MGEs.

10.1128/mSphere.00550-21.1 FIG S1 Graphical annotation of contigs harboring *cnf1/hlyCABD* and the location and direction of associated MGEs, including EC555 (I*S682* and IS*kpn37*) (a), EC488 (IS*682*, IS*kpn37*, IS*629*, IS*Ec10* (b), and cn_15494, and cn_4876 (c and d) EC672 truncated *cnf1* on two contigs (contigs no.67 and 72). Annotation has been done by Geneious Prime 2021.1.1. Download FIG S1, TIF file, 1.5 MB. Copyright © 2021 Pajand et al. 2021 Pajand et al. https://creativecommons.org/licenses/by/4.0/ This content is distributed under the terms of the Creative Commons Attribution 4.0 International license.

### Two genetic environments of *bla*_CTX-M-15_ detected in C1-M27 and the C2-subset.

**FIG 8:**
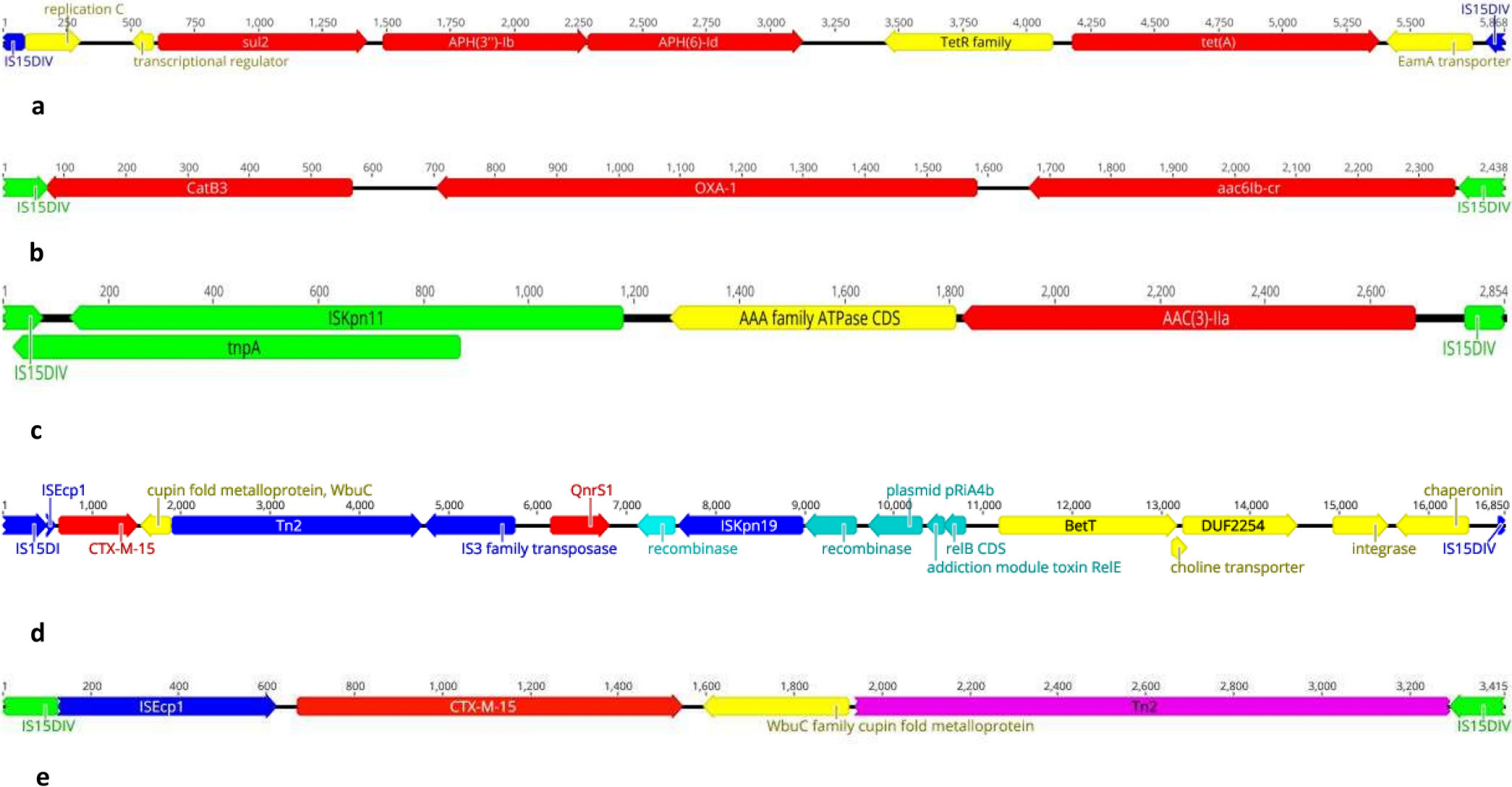
Genetic environment of the most common resistance genes among study isolates. (a to c) Contig harboring IS*15DIV*-*sul2*-*aph3Ib*-*aph6Id*-*tetA*-IS*15DIV* (a), IS*15DIV*-*catB3*-*bla*_OXA-1_-*aac6Ib-cr*-IS*15DIV* (b), and IS*15DIV*-IS*Kpn11*-*aac3IIa*-IS*15DIV* (c). (d and e) Two forms of the *bla*_CTX-M-15_ genetic environment. Annotation has been done by Geneious Prime 2021.1.1.

To predict the cotransfer of resistance genes with our sequencing results, the assembled genomes of all 64 strains were checked. The most common cocarriage of resistance markers was *aph3Ib*/*aph6Id*/*sul2*/*tetA* among C1-M27 and *aac6Ib-cr*/*bla*_OXA-1_/*catB3* among C2 strains; both were associated with IS*6* family transposase (IS*15DIV*) (Fig. 8a and b). Also, the gentamicin/tobramycin resistance marker, *aac3IIa*, was followed by AAA-family ATPase and a large insertion sequence IS*Kpn11*, which were detected among all C2 strains (Fig. 8c). Considering the *bla*_CTX-M-15_-carrying contigs, two different patterns were observed. First, the *bla*_CTX-M-15_ was followed by left-truncated IS*Ecp1* and preceded by cupin fold metalloprotein *WbuC*, a large Tn*2*, IS*3* transposase, *qnrS1*, IS*Kpn19*, and plasmid-related genes, including recombinase and addiction genes in three of the four C1-M27 strains. The *WbuC*, IS*Kpn19*, IS*3* transposase, and plasmid-related genes were located in the reverse direction (Fig. 8d).

The other genetic structure of *bla*_CTX-M-15_ gene observed on small contigs mostly among the C2-subset was IS*Ecp1* upstream of and in the same orientation as *bla*_CTX-M-15_, the reversed *wbuC* cupin fold metalloprotein, and a large Tn*2* transposon (Fig. 8e). In both identified genetic structures, *bla*_CTX-M-15_ was located between IS*15DI* (IS*6* family transposase), reversed *WbuC*, and a large Tn*2*.

## DISCUSSION

Along with our previous findings which have indicated a dynamic change toward the emergence of a subset harboring *cnf1/hlyA* virulence genes with aminoglycoside resistance phenotypes (16), we performed whole-genome sequencing of all ST131 isolates submitted from our health care center over 19 months. We performed sequence variation analysis on the globally dominant clade C isolates focusing on the *cnf1/hlyCABD*-positive ones. Using bioinformatics analyses, we examined the relatedness of positive strains and MGE association with the carriage of these virulence markers. Finally, we also applied genome annotation to identify the genetic environments of contigs carrying *cnf1/hlyCABD* and the resistance gene *bla*_CTX-M-15_.

The ST131 population in Semnan province was dominated by clade C, which is similar to results from a previous large global survey (17). Although C2 strains were found to be the most resistant subclade in our collection and were associated with *bla*_CTX-M-15_/*aac6Ib-cr*/*aac3IIa* and carriage of higher virulence markers, their prevalence was equal to the C1-subclade. The priority of the C1-subclade and specifically the C1-M27 subclade could be explained by these features: lower SNP differences and consequently higher clonality compared to C2 or even clade A strains, the great homogeneity of its plasmid content, and the virulence patterns (mostly virotype C) (18). However, the clonal displacement of different sublineages among ST131 population has been reported recently from Ireland in that the C1/CTX-M-14-producing subpopulation was displaced by the expansion of a genetically distinct C2 lineage referred to as C2-8 harboring chromosomally located CTX-M-15 in the *mppA* gene (17). Nevertheless, C1-M27 still has been reported as the dominant ST131 population from different countries such as Germany, France, and Japan (6, 19).

In the context of global ST131 genomes we found that two groups of Iranian strains, C1-M27 and C2-subset, which were equal in prevalence (*n* = 22 [34.3%]), have constituted distinct clusters. C1-M27 and C2 strains display two striking common features: first, producing cefotaxime/ceftazidime CTX-M hydrolyzing enzyme and, second, carriage of IncF-type plasmids encoding different resistance/virulence markers. While IncF-type plasmid among C2 was not as homogeneous as the C1-M27 subclade overall, focusing on the C2-subset revealed a unique pattern, namely, the F31/F36:A4:B1 replicon type. Again, most of the C2-subset displayed the same virotype (virotype E) showed a monophyletic origin and a common pattern of virulence/resistance markers. This C2-subset was also responsible for association of C2 population with higher virulence content and aminoglycoside resistance phenotype; nonetheless, this subpopulation seemed to be similar to other C2 strains overall in terms of its virulence/resistance repertoire. Therefore, it seems that possessing of virulence factors such as toxins (*cnf1*, hemolysin), invasins (*ibeB*, *tia/hek*), and adhesins (*hra*, *papC/GII*) likely contributes to the virulence potential of the C2-subset, and their overrepresentation in this subpopulation is related to the monophyletic nature of this subset.

The carriage of genomic island *GI-pheU* demonstrated among all *cnf1/hlyCABD*-positive strains (virotype E) and was not detected among EC958 or other C2 strains. Nonetheless, *GI-pheU* also carried other virulence markers, such as F17 fimbriae, *papE*, or *papA_F43*, which were absent among our strains. IS*Kpn37* and IS*682* were the most common detected MGEs flanking the *cnf1*/*hlyCABD* genes. However, two strains were found to display different patterns; in both, the *cnf1* gene was truncated and found on two contigs. Therefore, it seems that while *GI-pheU* acquisition plays a key role in the transfer of *cnf1/hlyCABD* genes among C2 strains, insertion sequences such as IS*682* are the second determinant in the transposition of *cnf1* inside the chromosome.

Diverse *bla*_CTX-M-15_ genetic environments have been reported among E. coli isolated all over the world (20). Structures containing the intact IS*Ecp1*, followed by *bla*_CTX-M-15_ and reversed *WbuC* cupin fold metalloprotein, have been identified in many different species (21). In our strain collection, two forms of CTX-M-15 genetic structure were observed, with the shared features of *bla*_CTX-M-15_, reversed *WbuC* metalloprotein, and Tn*2*. The unit, combined with the bounded two copies of IS*15DI*V (an IS*6* family member with a sequence and function similar to that of IS*26*), constitutes a composite transposon that is able to transfer by forming a circular molecule (22). The carriage of an identical IS*6*-mediated composite transposon has been reported mainly among E. coli plasmids (23). The other most common MGE in our strains, IS*Ecp1* (member of the IS*1380* family), has been shown to display two functions in association with *bla*_CTX-M-15_. First, it encodes a transposase that facilitates CTX-M-15 mobilization, and second, it promotes the weakly expression state of this ESBL gene by playing as a promoter (24). A histidine residue as a metal-binding ligand was identified in the WbuC family protein and has been reported to relate to O-antigen biosynthesis (20). The other RAST (Rapid Annotation using Subsystem Technology) annotation of the *WbuC* gene at this position was tryptophane synthase, due to the similar sequence protein that has been reported in Klebsiella spp. (20). Varied roles of *WbuC* have been attributed to this metalloprotein among different species; however, its specific function here with *bla*_CTX-M-15_ needs to be studied further. Another, less-reported *bla*_CTX-M-15_ genetic environment that was identified among three C1-M27 strains was IS*Kpn19*-*qnrS1-*IS*3* transposase-Tn*2*-*WbuC*-*bla*_CTX-M-15_ -IS*15DIV*. Similar structure units linked with IS*Kpn19*, instead of IS*26*, were seen in the chromosomes and plasmids of different bacterial species from China and other countries, suggesting that both IS*26* and IS*Kpn19* contribute to the circulation of a *qnrS1*- and *bla*_CTX−M−15_-carrying unit between chromosomes and plasmids of different bacterial species (23).

In summary, our study is notable for examining ST131 clone in an undersampled geographical region. This allowed us to identify the local epidemiology of this high-risk clone. Our findings highlight a subpopulation harboring *cnf1/hlyCABD* genes combined with up to seven MGEs (mostly IS*682*/IS*Kpn37*) within the horizontally acquired genomic island *GI-pheU*. The diversity observed among ST131 lineages and resistance/virulence markers emphasizes the role of surveillance strategies to identify ST131 subpopulations, plasmids, and MGEs. Focused attention on successful subpopulations could be helpful to identify the dynamics of emergence and their spread and control the epidemic resistance of E. coli.

## MATERIALS AND METHODS

### Bacterial collection, sequencing, and antibiotic susceptibility testing.

A total of 72 E. coli ST131 isolates from a single tertiary referral hospital (Kosar) covering a >120,000 population of Semnan province-Iran were collected. Isolates were collected as part of standard care for admitted patients from extraintestinal specimens, including urine, blood, wound, and respiratory samples. The demographic data are shown in Table 1.

To extract genomic DNA, an Invitrogen Easy-DNA kit was used, and the DNA concentrations were determined using a Qubit dsDNA BR assay kit (Invitrogen). According to the Illumina protocol, library preparation was conducted, and sequencing was performed on Illumina NextSeq and MiSeq platforms using 150-bp paired-end reads. SPAdes was used to *de novo* assemble raw reads (25).

Antibiotic susceptibility testing was performed by using a standard disk diffusion method for the 16 antimicrobial agents. The results were interpreted according to Clinical and Laboratory Standard Institute (CLSI) guidelines (26). Isolates with intermediate or resistant phenotypes to a given antimicrobial agent were considered nonsusceptible. Multidrug resistance (MDR) was defined as resistance to at least one representative of three or more antimicrobial classes (27). An extended-spectrum beta-lactamase phenotype was confirmed by using a phenotypic combined disk test according to CLSI recommendations (26).

### Genome sequence analysis.

Assembled sequences were analyzed using *in silico* bioinformatics tools to confirm the serotype, sequence type, plasmid replicons, acquired antimicrobial resistance genes/fluoroquinolone resistance-associated mutations, virulence genes, plasmid MLST (pMLST) using the following pipelines: SeroTypeFinder 2.0 (12), MLST 2.0 (28), PlasmidFinder 2.1 (29), ResFinder 4.1 (30), VirulenceFinder 2.0 (31), and pMLST 2.0 (29) available from CGE. For virulence genes, the VFDB database was also used as the web interface (32). The raw-FastQ reads (*n* = 289 genomes) from a recently published comprehensive study were downloaded from European Nucleotide Archive (ENA) (17). Furthermore, 25 ST131 raw reads recently reported from Singapore were also included (15). In total, 314 ST131 short read data sets were subjected to the same pipeline mentioned above for *de novo* assembly, and the generated assemblies were processed using the databases described above. The genome annotation of some related resistance/virulence harboring contigs was performed with RAST, and .gbk and or Excel files format were downloaded. The association of resistance/virulence markers with MGEs was investigated by MGEFinder 1.0 (available from CGE) (33). The genetic environments of contigs harboring the *cnf/hly* virulence and *bla*_CTX-M-15_ resistance genes were graphically annotated by Geneious Prime 2021.1.1, and insertion sequence positions were manually curated using ISfinder BLAST results (34). BLAST Ring Image Generator (BRIG) was used to determine the similarity of genomes to ST131 genomic islands (35).

Reference based analysis was used to create the phylogenetic tree using reference strain EC958 (36). The paired-end reads were mapped to the reference genome using Burrows-Wheeler Aligner (BWA), version 0.7.2 (37). SNP calling was done using the mpileup module in SAMTools version 0.1.18. The SNP selection was based on the following criteria: (i) a distance of minimum 15 bp between each SNP (pruning), (ii) an average depth of minimum 10%, (iii) a quality of mapping of >30, (iv) an SNP quality of >20, and (v) the exclusion of all indels. Qualified SNPs from each genome were concatenated to a single alignment corresponding to the position of the reference genome. The parsimony tree construction based on PhyML was used for concatenated sequences (38) with an HKY85 substitution model and 100 bootstrap replicates. The generated phylogenetic tree was visualized and labeled with the iToL tool. Based on cgMLST allelic mismatch between strains, a minimum spanning tree (MST) was designed by uploading the assembled genomes to Ridom SeqSphere (Ridom GmbH, Munster, Germany). Clonal clustering was defined based on a maximum of 10 allelic differences.

Clades A, C1, and C2 were identified based on matching strain identifiers with the previous report by Matsumura et al. (39). *H*30Rx was defined based on the specific single nucleotide variant *ybbW*(G723A), as identified by BLASTn (40). Isolates were defined as ExPEC if they were positive for ≥2 for any *pap*, any *sfa* or *foc*, any *afa* or *dra*, or any *kps* or *iutA* (41). Virotypes were determined based on the virulence genes scheme (42). Isolates with ≥1 of the enteroaggregative E. coli (EAEC)-associated genes *aggR*, *aatA*, *aaiC*, and *aaP* were molecularly qualified as EAEC (43).

### Statistical analysis.

The IBM-SPSS statistics 22 was used for all statistical analysis. Bivariate analyses were performed by using a chi-square test for categorical variables. All *P* values were two-sided.

### Data availability.

Raw sequence data have been submitted to the European Nucleotide Archive under study accession number PRJEB46895.

10.1128/mSphere.00550-21.2 DATA SET S1 Excel file dataset. Sheet 1 includes the data of public E. coli ST131 clade C strains, country and source of isolation, and genotyping results. Sheet 2 includes the study isolates (Iranian isolates) and genotyping results. Download Data Set S1, XLSX file, 0.03 MB. Copyright © 2021 Pajand et al. 2021 Pajand et al. https://creativecommons.org/licenses/by/4.0/ This content is distributed under the terms of the Creative Commons Attribution 4.0 International license.
