# Supplementary figures and images for: Arrangements of Mobile Genetic Elements among Virotype E Subpopulation of Escherichia coli Sequence Type 131 Strains with High Antimicrobial Resistance and Virulence Gene Content
Source: mSphere. 2021 Aug 25;6(4):e00550-21. doi: 10.1128/mSphere.00550-21 (PMC8386418; doi:10.1128/mSphere.00550-21)

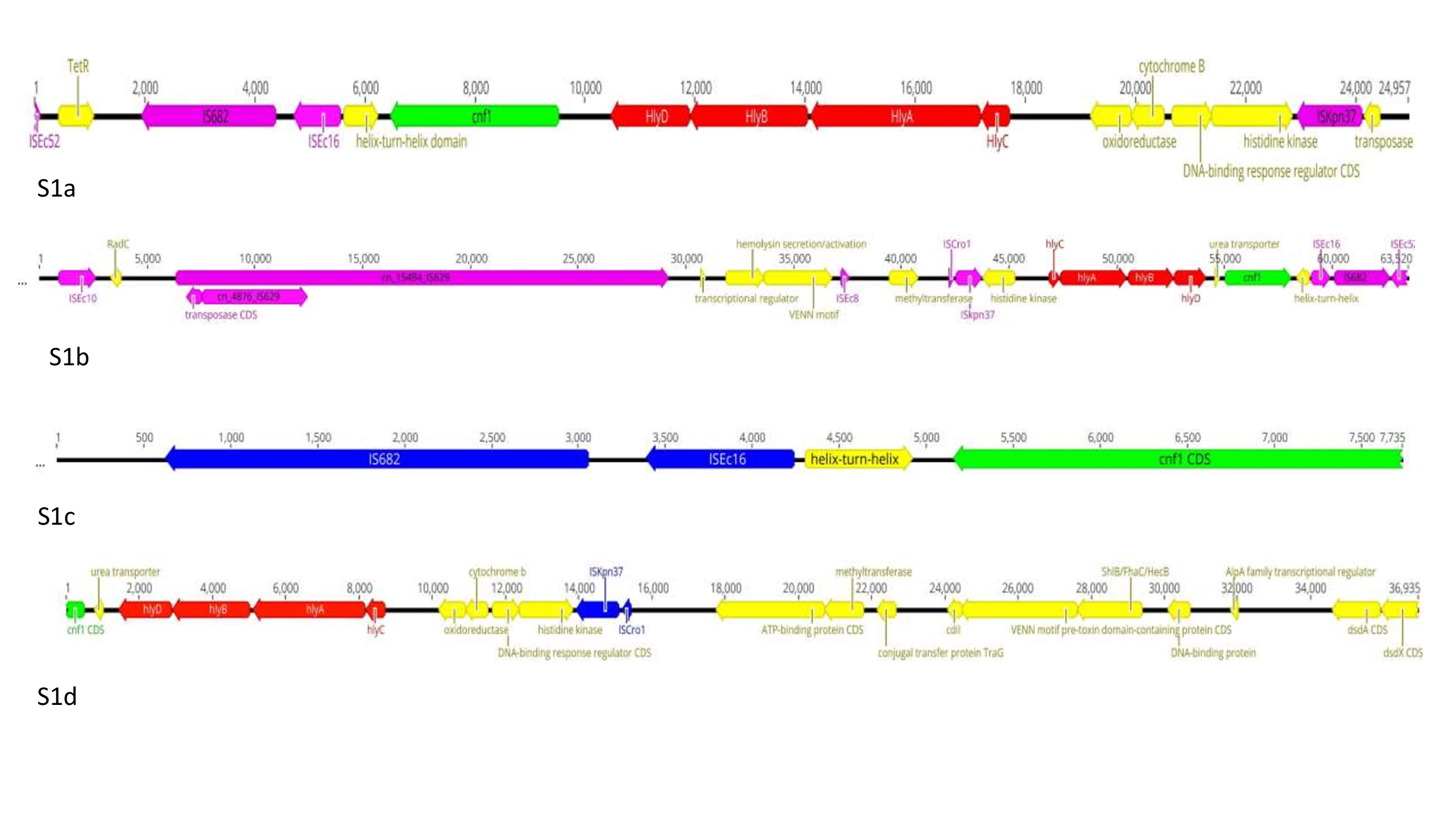

Supplement: FIG S1 [file msphere.00550-21-sf001.tif]
